# Role of Methyl thiobutyrate to *Botrytis cinerea* on cucumber

**DOI:** 10.3389/fpls.2025.1551274

**Published:** 2025-04-08

**Authors:** Nv Chang, Rui Liu, Cuihua Lu, Yuqing Lai, Qian Xu, Yuhong Yang, Yan Li, Jian Ling, Bingyan Xie, Wenchao Zhao, Zhenchuan Mao, Jianlong Zhao

**Affiliations:** ^1^ Beijing Key Laboratory for Agricultural Application and New Technique, College of Plant Science and Technology, Beijing University of Agriculture, Beijing, China; ^2^ State Key Laboratory of Vegetable Biobreeding, Institute of Vegetables and Flowers, Chinese Academy of Agricultural Sciences, Beijing, China

**Keywords:** *Botrytis cinerea*, cucumber, Methyl thiobutyrate, induced resistance, biocontrol agent

## Abstract

**Introduction:**

*Botrytis cinerea* is a major agricultural pathogen that causes significant economic Q7 losses worldwide, affecting various crops, including cucumbers. Developing environmentally sustainable control strategies for this pathogen is crucial. Methyl thiobutyrate (MTB), a small organic molecule identified in the volatile organic compounds (VOCs) of biocontrol bacteria, has demonstrated potential in inhibiting *B. cinerea* both *in vitro* and *in vivo*.

**Methods:**

In this study, the efficacy of MTB against cucumber gray mold disease was examined by assessing the *in vitro* and *in vivo* activities of MTB against *B. cinerea* and analyzing the transcriptomic data from MTB-treated cucumber leaves infected with *B. cinerea*.

**Results and discussion:**

This study shows that a 2 mg/mL solution of MTB inhibits *B. cinerea* growth by 98.6% in vitro. In vivo, MTB effectively reduces *B. cinerea* infection in cucumbers, alleviates necrotic damage in leaf tissues, and significantly reduces disease severity. Transcriptomic analysis reveals that MTB activates the plant immune responses by modulating key MAPK cascade signaling genes and upregulating basal defense genes, including chitinase, pectinase, and lignin biosynthesis genes. Furthermore, MTB influences the signaling pathways of salicylic acid (SA), jasmonic acid (JA), and ethylene (ET), resulting in the upregulation of genes such as peroxidase (POD), phenylalanine ammonia-lyase (PAL), lipoxygenase (LOX), and ethyleneresponsive transcription factors (ERFs). These results demonstrate the potential of MTB as an effective biocontrol agent against *B. cinerea* and provide valuable insights into its underlying mechanisms of action.

## 1 Introduction

Cucumber is an important vegetable crop in China, with a cultivation area of 1.044 × 10^6^ hectares and a production volume of 5.624 × 10^7^ metric tons, accounting for 52.7% and 74.8% of the global production and cultivation area, respectively (Ezazi et al., 2021). Gray mold disease, caused by *Botrytis cinerea*, is a severe fungal disease in cucumber, primarily affecting the flowers, fruit stems, and leaves, leading to necrosis and the formation of gray mold (Liu et al., 2020). This disease is characterized by low-temperature and high-humidity conditions. With the rise of protected agriculture, the optimal conditions for *B. cinerea*—low temperatures, high humidity, and low light—have increasingly exacerbated the disease’s impact. In mild cases, the disease can reduce yields by 20% to 30%, while in severe cases, it can result in losses exceeding 50% or even complete plant death (Legard et al., 2000).

Currently, chemical control remains the primary method for managing *B. cinerea*. However, *B. cinerea* is known for its high resistance potential, having developed resistance to a variety of fungicides with different modes of action, including benzimidazoles, dicarboximides, phenylpyridines, carbamates, amides, demethylation inhibitors (DMIs), phenylpyrroles, and sterol biosynthesis inhibitors (Elad et al., 2006; Zhang et al., 2020). The most effective control measures are implemented before the disease onset or during its early stages. While chemical agents are effective in prevention, their overuse leads to issues such as resistance, resurgence, and pesticide residues—collectively known as the 3R problems—which raise significant environmental and health concerns (Tang et al., 2022). The widespread use of chemical fungicides not only contributes to severe environmental pollution but also accelerates pathogen resistance development. Resistance breeding has become a critical strategy to combat *B. cinerea*, focusing on the evaluation and screening of disease-resistant resources using reliable resistance assessment techniques, molecular marker-assisted breeding, and the development of resistant cultivars. However, in China, research on disease-resistant cucumber varieties is limited in areas such as genetic pattern identification, gene localization for *B. cinerea* resistance, and gene cloning (Li H. et al., 2023). Furthermore, the long breeding cycles and limited resources have slowed the progress of resistance breeding. The underlying molecular mechanisms facilitating broad host-range necrotrophy have not been well defined to date (Chen et al., 2022).

Induced resistance is an emerging strategy to control *B. cinerea* by enhancing the plant’s immune response. This mechanism, often referred to as systemic acquired resistance (SAR) or plant immunity, represents a rapid and robust defense reaction triggered by specific elicitors. These elicitors can be biological or chemical in nature, and their application induces a series of defensive responses in the plant, such as oxidative bursts, reinforcement of cell walls, and the production of antimicrobial compounds (Walters et al., 2013). Plant resistance mechanisms, including induced systemic resistance (ISR) and SAR, modulate the biosynthesis of signaling molecules like salicylic acid (SA) and jasmonic acid (JA), which are crucial for defending against necrotrophic pathogens and herbivores (Romera et al., 2019).

Volatile organic compounds (VOCs), produced during microbial secondary metabolism, are key elicitors that induce resistance in plants. VOCs serve as antimicrobial agents for microorganisms, as well as long-distance signaling molecules in plant-microbe interactions (Tina Netzker, 2020). For instance, β-aminobutyric acid (BABA), a non-protein amino acid, has shown promise in enhancing disease resistance in crops such as chili (Safaie Farahani and Taghavi, 2017), tomato (Wilkinson et al., 2018), and apple (Ron Porat, 2003). Additionally, BABA has been shown to induce resistance in tomatoes against *B. cinerea* and in apples against blue mold, while maintaining fruit quality during storage (Li Y. et al., 2023).

Methyl thiobutyrate (MTB), a small sulfur-containing organic molecule, is slightly water-soluble and has some volatility. It has been identified as a major component in the volatile organic compounds of biocontrol bacteria (Xu et al., 2015). Through gas chromatography/mass spectrometry (GC/MS), 21 components were identified from the VOCs produced by *Bacillus cereus* Bc-cm103, with MTB (CAS NO 2432-51-1) accounting for 30.29% of the total VOCs (Yin et al., 2021). Recent studies have demonstrated that *Bacillus cereus* N4, isolated from orchard soil, can significantly inhibit the sporulation and mycelial growth of fruit molds. Following fumigation with VOCs, the incidence rate and lesion diameter on peach fruits were significantly lower compared to controls, with MTB being one of the primary antifungal compounds identified (Shi et al., 2024). Other compounds, such as 3-methyl-1-butanol produced by *Aureobasidium* species can inhibit the growth of *B. cinerea* in tomatoes and grapes (Di Francesco et al., 2020). Interestingly, 3-methyl-1-butanol and MTB are widely used in the preparation of food flavors, with common applications in foods such as cheese, cocoa, and cream, and can even be used to highlight the quality and freshness of food (Sampaio et al., 2011). Additionally, 3-methyl-1-butanol produced by *Phaeosphaeria nodorum* can inhibit the growth of *Monilinia fructicola*, the pathogen causing brown rot in peaches (Pimenta et al., 2012). Evidence have also demonstrated VOCs, such as MTB, produced by bacteria like *Achromobacter xylosoxidans*, exhibit nematicidal activity against *Meloidogyne incognita* and *Caenorhabditis elegans*. Recently, it was confirmed that *Bacillus cereus* can repel root-knot nematodes and induce plant resistance to these pests by producing volatile secondary metabolites containing MTB, thus playing a role in the management of root-knot nematodes (Yin et al., 2021). Given its broad-spectrum antimicrobial properties and potential to induce resistance, MTB could be an effective agent for controlling *B. cinerea* in cucumbers. This study focuses on evaluating the inhibitory effects of MTB on *B. cinerea* in cucumbers both *in vitro* and *in vivo*. We also investigate whether MTB can induce plant resistance to *B. cinerea*, providing a foundation for the potential use of MTB as a biocontrol agent in the management of gray mold disease in cucumbers.

## 2 Methods

### 2.1 Main materials

The *Botrytis cinerea* strain, responsible for cucumber gray mold disease, was obtained from the Disease Group of the Vegetable and Flower Institute, Chinese Academy of Agricultural Sciences (IVF-CAAS), and cultured on Potato Dextrose Agar (PDA) medium. The strain of *Botrytis cinerea* was isolated from cucumber plants showing gray mold symptoms in local fields. This strain has been studied for its pathogenicity in the laboratory and reflects the major genotypes affecting cucumber crops in this region. Moreover, the strain has been thoroughly characterized at the molecular level, including sequencing and analysis of its genome. This strain exhibits strong pathogenicity locally, leading to significant reductions in cucumber yield and quality. In our previous concentration gradient experiments, the optimal concentration of the *Botrytis cinerea* spore suspension was determined to be 10^2^ spores/ml. At this concentration, the application of MTB significantly inhibited gray mold disease in cucumber plants. However, the experimental results indicated that when the concentration of the spore suspension exceeded 10^2^ spores/mL, the effect on gray mold disease was not significant regardless of whether MTB was applied or not. This finding suggests that at a concentration of 10^2^ spores/mL, the inhibitory effect of MTB is optimal, and higher concentrations do not further enhance the efficacy of MTB in disease control.

Cucumber variety Zhongnong No.6 (*Cucumis sativus*) was provided by Prof. Shenping Zhang (IVF-CAAS). The seeds were surface-sterilized by soaking in a 0.5% sodium hypochlorite solution for 3 minutes, rinsed three times with sterile water, and then germinated in the dark at 28°C for 1 day. Methyl thiobutyrate (MTB) was purchased from Beijing Dehang Wuzhou Technology Co., Ltd.

### 2.2 The inhibitory effect of MTB to *B. cinerea in vitro*

To assess the direct inhibitory effect of MTB on *B. cinerea* mycelium and spores, MTB was prepared in concentrations of 2 mg/mL, 0.2 mg/mL, and 0.02 mg/mL in distilled water.

Antimicrobial Experiment: PDA plates containing MTB were prepared to achieve final concentrations of 2 mg/mL, 0.2 mg/mL, and 0.02 mg/mL. A 0.7 cm agar disk of *B. cinerea* was placed at the center of each plate and incubated in the dark at 28°C for 2–7 days. The fungal colony diameter was measured, and the inhibition rate was calculated. Distilled water served as the negative control, while hygromycin (1 mg/mL) was used as the positive control. Five plates per treatment were used, and the experiment was repeated three times. The inhibition rate was calculated according to previous report (Hossain, 2024), using the formula:


Inhibition rate(%)=(1−Diameter of Colony/Diameter of Treated Colony)×100%

### 2.3 Analysis of MTB-induced plant immune signaling pathways

To determine if MTB triggers the SA and JA signaling pathways, RT-qPCR was used to assess the expression of pathogenesis-related (PR) gene (*PR-1*) and protease inhibitors (*PIs*) in cucumber leaves. Cucumber seedlings at the one-leaf stage were treated with a spray application of 0.02 mg/mL MTB (3 mL per plant). Total RNA was extracted from the treated leaves using the RNAprep Pure Plant Total RNA Extraction Kit (Tiangen Biotechnology, Beijing, China). After quantifying RNA concentrations, reverse transcription was performed using the PrimeScript™ RT Reagent Kit with gDNA Eraser (RR047A, BaoLinKe, Beijing). Primers for qPCR were designed using the Primer3Plus online tool (https://www.primer3plus.com/). Real-time PCR was performed using SYBR Green I, and relative gene expression was calculated using the ΔΔCt method (Zhou et al., 2023). Primers are provided in **Supplementary Table S4**.

### 2.4 The efficacy of MTB to *B. ciner*ea *in vivo*

To evaluate the inhibitory effect of MTB on *B. cinerea* infection in detached cucumber leaves, Zhongnong No.6 cucumber plants were grown under normal temperature conditions. At the one-leaf stage, the first true leaves were harvested, rinsed with distilled water, and gently dried with filter paper. Sterilized petri dishes containing two layers of filter paper moistened with water were prepared, and the leaves were placed inside.

A spore suspension of *B. cinerea* (1 × 10^2^/mL) was prepared by washing conidia from PDA plates (7 days old). Detached leaves were treated with 0.02 mg/mL MTB for 1 day before inoculation with the *B. cinerea* spore suspension by spray. Six leaves per treatment were used, with the temperature set to 20°C. Disease progression was monitored 4-6 days post-inoculation, and the disease index was calculated. Disease severity was graded as follows: Grade 0: no lesions; Grade 1: lesions covering 1%-10% of the leaf area; Grade 3: lesions covering 11%-25%; Grade 5: lesions covering 26%-50%; Grade 7: lesions covering 51%-75%; Grade 9: lesions covering >75% (Willocquet et al., 2023). The disease index was calculated using the formula:


Disease Index=(∑(Disease Grade×Number of Leaves with that Grade))/(Total Number of Leaves Rated×Maximum Disease Grade)×100

### 2.5 Pathological observation

To investigate the effect of MTB on plant cell death, Trypan Blue staining was employed (Kerschbauma et al., 2021). The seedlings were treated with the *B. cinerea* spore suspension (1 × 10^2^/mL) and observed for infection symptoms at 1, 2, 3, and 7 days post-inoculation. Distilled water was used as the control. The leaves were stained with Trypan Blue to visualize cell death. The staining solution was prepared by boiling 10 mL lactic acid, 10 mL glycerin, 10 g phenol, and 10 mg Trypan Blue in 10 mL distilled water for 10 minutes. After staining, the leaves were decolorized in a solution of ethanol and lactophenol until the background turned white (de Oliveira et al., 2016).

### 2.6 Pot test of MTB to *B. cinerea* of cucumber

For pathogen detection of *B. cinerea in vivo*, one-leaf stage seedlings of Zhongnong No.6 cucumber were treated with 3 mL of 0.02 mg/mL MTB, and the control group was treated with sterile water. *B. cinerea* spores (1 × 10^2^/mL) were prepared, and plants were inoculated by spray. Samples were collected at 0 hours (before inoculation), 12 hours, 24 hours, and 48 hours for RT-qPCR to assess the expression levels of *B. cinerea* under different treatments.

For potted plant efficacy analysis, Zhongnong No.6 cucumber plants were treated with 0.02 mg/mL MTB at the one-leaf stage (3 mL per plant). *B. cinerea* spores (1 × 10^2^/mL) were inoculated by spray. Disease severity was monitored at 4, 7, and 11 days post-inoculation. Disease index calculations followed the same method as described for the detached leaf inhibition test.

### 2.7 MTB-induced cucumber transcriptome analysis

To clarify the regulatory mechanism of MTB in cucumber’s defense against *B. cinerea*, a comparative transcriptome analysis was conducted to examine the differentially expressed genes before and in MTB treatment. Cucumber leaves were treated with 0.02mg/ml MTB at the one-leaf stage, and 12 hours later, a spore suspension of *B. cinerea* (1x10^2^/ml) was sprayed onto the leaves. Samples were collected at 12 hours, 24 hours, and 4 days post-inoculation, with equal amounts of samples from the three time points mixed to form the MTB treatment samples, and each sampling was repeated three times for sequencing. In the experiment, an equal amount of distilled water was used as a control treatment for *B. cinerea* inoculation, and cucumber leaf samples were taken for three parallel sequencing runs. Sequencing was performed by Personal Biotechnology Co., Ltd (Shanghai, China). The clean reads were deposited in the NCBI Sequence Read Archive under accession number PRJNA1194377. Subsequent analyses were conducted using Genescloud (https://www.genescloud.cn/home).

Raw data in FASTQ format were obtained, followed by quality filtering. The reads were aligned to the reference genome using TopHat2 and HISAT2. Based on the alignment results, the expression level of each gene is calculated. For identifying the differentially expressed genes (DEGs), genes are filtered using the following criteria: a significant expression difference with |log2FoldChange| greater than 1, and a *P*-value less than 0.05. The number of up regulated and down regulated differentially expressed genes is then statistically determined.

Differential expression was analyzed using |log2FoldChange| > 1 and a P-value< 0.05. Gene Ontology (GO) enrichment and KEGG pathway analyses (Kyoto Encyclopedia of Genes and Genomes, http://www.kegg.jp/) were performed to identify biological functions and pathways associated with the DEGs (Chen et al., 2019; Wu et al., 2021).

### 2.8 Statistical methods

The significance of differences between two groups was evaluated using Student’s t-test. For assessments involving multiple comparisons, a one-way ANOVA method was conducted, followed by Tukey *post hoc* test to determine the differences. All statistical analyses were executed utilizing GraphPad Prism software, version 8.3.0.

## 3 Results

### 3.1 Inhibition of *Botrytis cinerea* Growth by MTB *in vitro*

**Figure 1:**
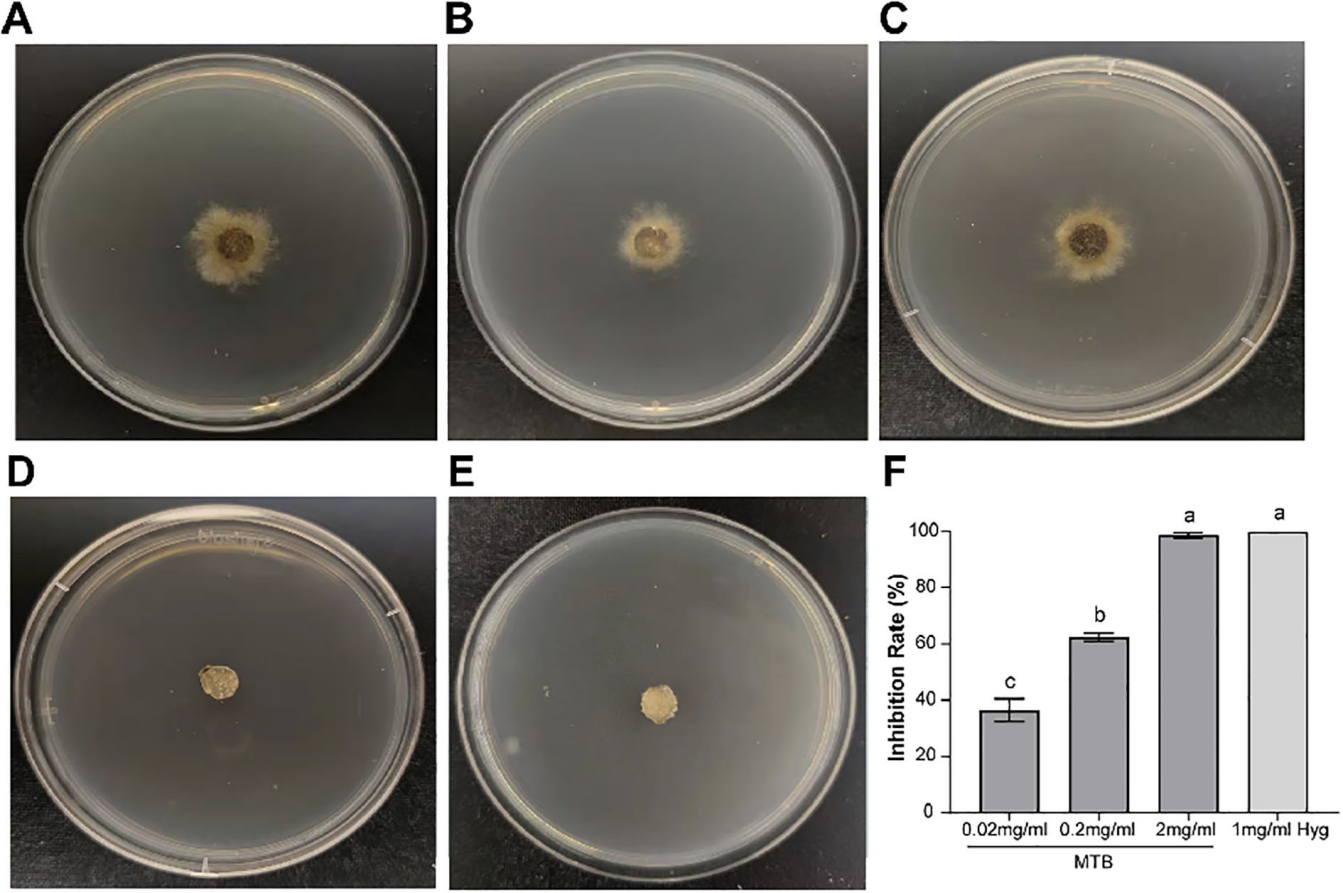
Direct inhibitory effect of MTB on *Botrytis cinerea*. **(A)** Growth of *B. cinerea* colonies on PDA plates after 2 days of cultivation. **(B)** Growth of *B. cinerea* colonies on PDA plates treated with 0.02 mg/ml MTB. **(C)** Growth of *B. cinerea* colonies on PDA plates treated with 0.2mg/ml MTB. **(D)** Growth of *B. cinerea* colonies on PDA plates treated with 2mg/ml MTB. **(E)** Growth of *B. cinerea* colonies on PDA plates treated with 1mg/ml Hyg. **(F)** Bar chart shows inhibition rates of *B. cinerea* under different treatments. The columns and bars represent the mean ± standard deviation of three biological replicates for each treatment. Different letters indicate significant differences (*P*<0.05, one-way ANOVA followed by Turkey analysis).

To investigate the direct inhibitory effect of MTB at different concentrations to the mycelium and spores of *B. cinerea*, the agar confrontation method was used to observe the growth of *B. cinerea* colonies. It was found that the control clone produced a lot of white mycelium on the second day (**Figure 1A**), while the MTB treatment groups showed a significant reduction in colony diameter as the concentration increased (0.02mg/ml, 0.2mg/ml, 2mg/ml) (**Figures 1B–D**), and the colony morphology of the 2mg/ml MTB treatment group was similar to that of the 1mg/ml hygromycin treatment group (**Figure 1E**). Meanwhile, the inhibition rates of MTB treatment groups were 36.4% ± 4.0%, 62.4 ± 1.5%, and 98.8 ± 1.0% respectively (**Figure 1F**), with the high concentration of MTB approaching the 100% inhibition rate achieved with hygromycin treatment.

### 3.2 MTB-mediated induction of SA and JA signaling pathways

**Figure 2:**
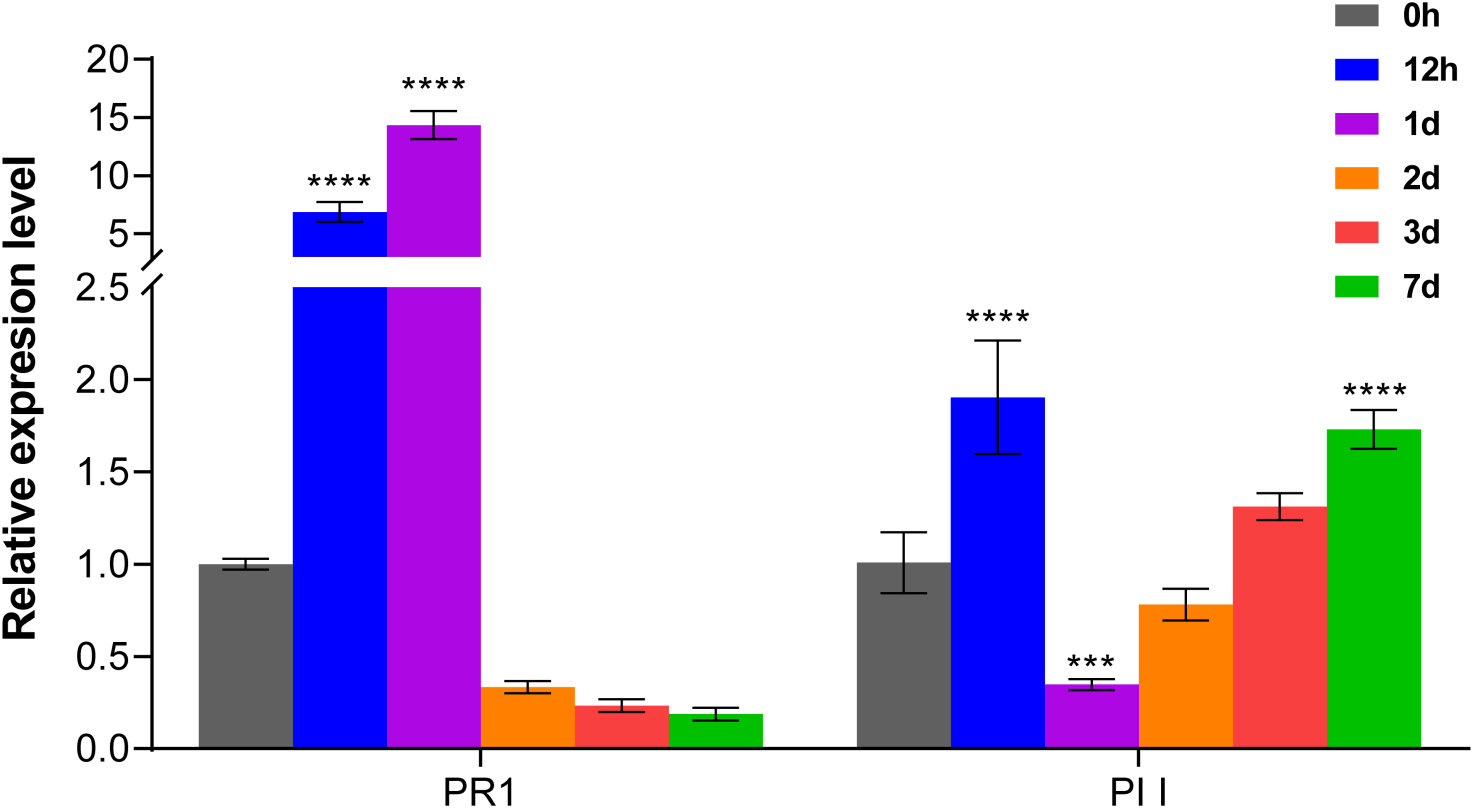
MTB treatment influences the expression of immune-related marker genes in cucumber. The relative expression levels of *PR1* (pathogenesis-related protein 1, salicylic acid signaling) and *PI I* (protease inhibitor 1, jasmonic acid signaling) marker genes were quantified using the 2^−ΔΔCt^ method, with the cucumber *CsActin* gene as an internal reference. Data are presented as mean ± SD from three independent experiments, and asterisks indicate statistically significant differences using Student’s *t*-test (*** p< 0.001,**** p< 0.0001).

In order to investigate the role of salicylic acid (SA) and jasmonic acid (JA) signaling pathways in MTB-mediated immunity, we examined key pathogenesis-related protein genes, *PR-1* (associated with SA signaling) and protease inhibitors (*PIs*, related to JA signaling), after MTB treatment. PR1 is a critical regulator in the plant’s systemic acquired resistance (SAR) pathway (Van Loon and Van Strien, 1999), while PIs serve as marker genes that enhance plant defense responses via the JA signaling pathway (Martinez et al., 2016). RT-qPCR analysis revealed that MTB treatment rapidly induced *PR1* gene expression in cucumber leaves, with a peak of 14.3-fold on day 1, followed by a quick decline. In contrast, *PII* gene expression exhibited a slower response, with higher expression levels observed at 12 hours and again at 7 days post-treatment. These results confirm that MTB treatment play a role in both the SA and JA signaling pathways, leading to upregulated immune responses in cucumber plants (**Figure 2**).

### 3.3 MTB inhibited the pathogenicity of *B. cinerea* leaves *in vitro*

**Figure 3:**
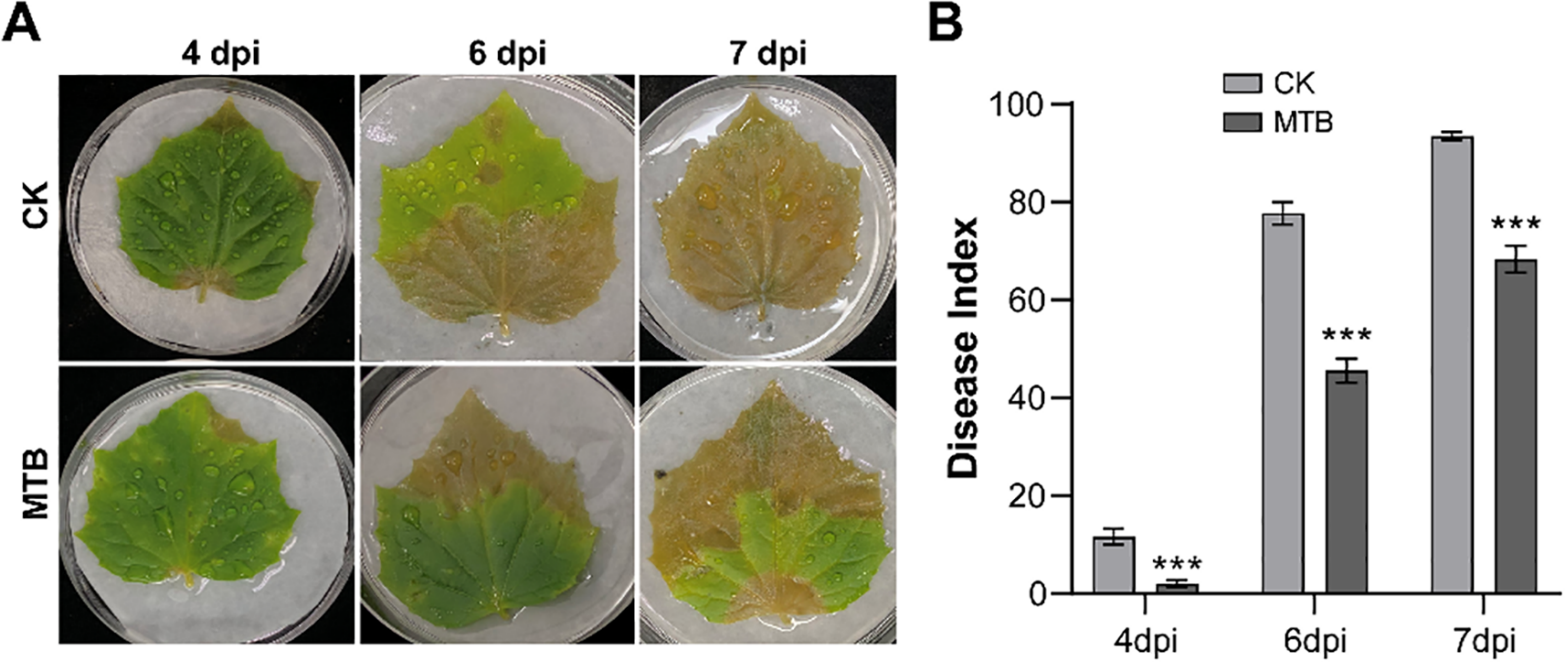
The control effect of MTB on gray mold disease of cucumber leaves *in vitro*. **(A)** Symptoms on detached cucumber leaves inoculated with *Botrytis cinerea* under different treatments on 4, 6, and 7 days post-inoculation (dpi). **(B)** The disease index of cucumber gray mold under different treatments. Error bars denote standard deviations from three independent experiments, and asterisks indicate statistically significant differences using Student’s *t*-test (*** *p*< 0.001).

In order to assess the inhibitory effect of MTB on *B. cinerea* infection in detached cucumber leaves, 3 ml of MTB (0.02 mg/ml) or an equal volume of distilled water (control) were sprayed onto the leaves. One day after treatment, the leaves were inoculated with a suspension of *B. cinerea* spores. Disease symptoms were monitored on days 4, 6, and 7, and significant differences between MTB-treated and control leaves were observed (**Figure 3A**). Disease severity was quantified by calculating the disease index (**Figure 3B**). On day 4, the control group showed water-soaked lesions with a disease index of 11.6 ± 1.6, while the MTB-treated group had a significantly lower disease index of 2.0 ± 0.6, indicating a milder disease condition. By day 6, both groups exhibited widespread disease, but the lesion area in the MTB-treated group was notably smaller than in the control group. The disease index in the treated group was 45.5 ± 2.0, significantly lower than the control group’s 77.7 ± 2.2. On day 7, the control leaves were fully infected, with a disease index of 93.5 ± 0.8, while the MTB-treated leaves had a disease index of 68.3 ± 2.3. These results demonstrate that MTB treatment effectively inhibits *B. cinerea* infection and reduces disease severity in detached cucumber leaves.

### 3.4 MTB reduces leaf tissue necrosis

**Figure 4:**
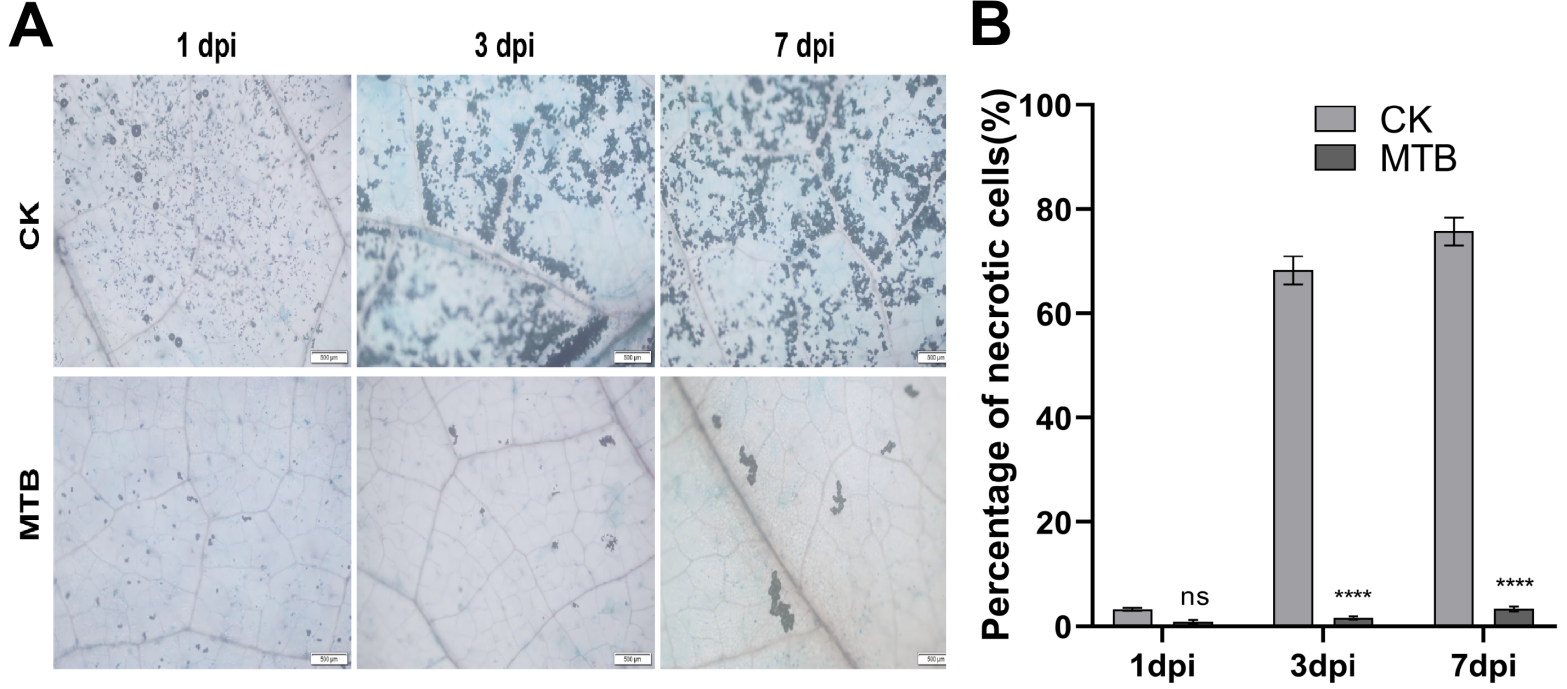
MTB treatment reduces necrosis in cucumber leaves infected with *Botrytis cinerea* compared to the control. **(A)** Cucumber leaves were treated with MTB (0.02 mg/ml) or distilled water (CK), followed by inoculation with *B. cinerea*. Propidium iodide staining was performed, and microscopy images were captured at 1, 3, and 7 days post-inoculation. Blue spots indicate dead cells, stained due to compromised membrane integrity. **(B)** Percentage of necrotic cells in cucumber leaves under different treatments. Error bars denote standard deviations from three independent experiments, and asterisks indicate statistically significant differences using Student’s *t*-test (**** p< 0.0001, ns indicates no significant difference.).

Trypan Blue staining was used to assess cell viability, as the dye cannot penetrate healthy cell membranes. Consequently, viable cells remain unstained, while dead or damaged cells with compromised membrane integrity are stained (**Figure 4A**). The percentage of necrotic cells was quantified using ImageJ software (v1.53). Compared with the control group, the MTB-treated group exhibited a 72% reduction in cell death at 7dpi (**Figure 4B**), indicating the observed mitigation of tissue damage. On the first day post-inoculation, both the control and MTB-treated groups displayed distinct blue necrotic spots. By day 3, the control group exhibited extensive staining, reflecting a significant increase in necrotic cells and a corresponding decrease in viable cells. In contrast, the MTB-treated group showed much less staining, with fewer necrotic cells, indicating a reduction in tissue damage compared to the control group. On day 7, the control group displayed further intensification of staining, consistent with the progression of *B. cinerea* infection and increased cell death. In the MTB-treated group, the staining was much less pronounced, with fewer dead cells present. These results suggest that MTB treatment effectively reduces leaf tissue necrosis.

### 3.5 MTB reduced the pathogen quantity of *B. cinerea*

**Figure 5:**
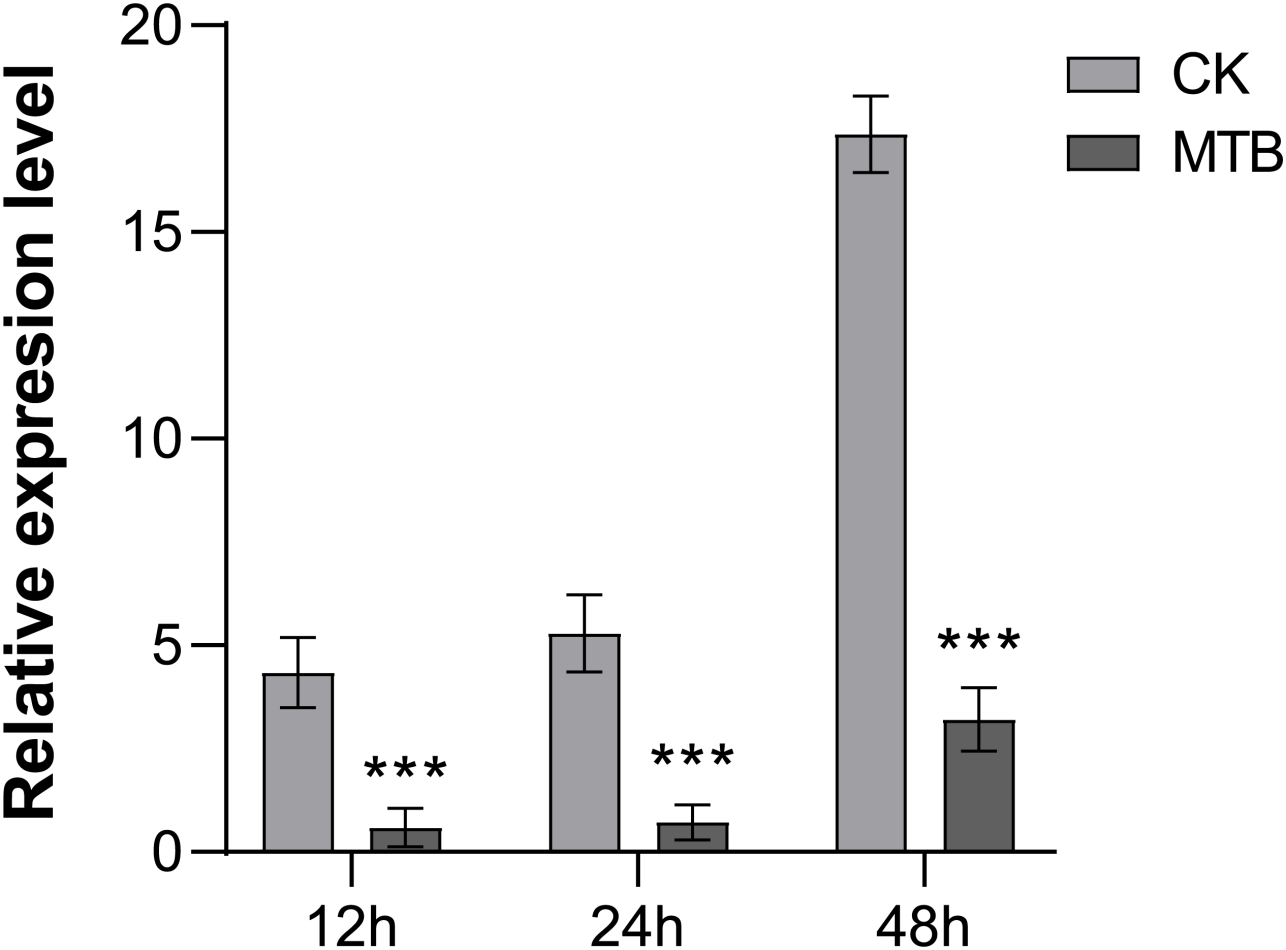
MTB treatment enhances the gray mold disease resistance of cucumber leaves. The fungal biomass was determined by qPCR of *Botrytis cinerea Bcactin* gene against the cucumber *CsActin* gene. Data are presented as mean ± SD from three independent experiments, and asterisks indicate statistically significant differences using Student’s *t*-test (*** *p*< 0.001).

To assess the impact of MTB on the intracellular growth of *B. cinerea*, we employed reverse transcription quantitative polymerase chain reaction (RT-qPCR) to quantify the pathogen’s DNA content within treated and control plant leaves. The analysis showed significant differences between the treated and control groups, with notable changes observed 12 to 24 hours after inoculation with *B. cinerea*. The pathogen count in the control group was five times higher than in the MTB-treated group. At 48 hours, the quantity of the pathogen increased in both treatments, but the difference between them became more significant, which the relative content of *B. cinerea* in MTB treatment was 3.21 times, while in the control (CK) it was 17.37 times (**Figure 5**). These results further demonstrates that as time increases, the inhibitory effect of MTB on *B. cinerea* increases with time.

### 3.6 MTB effectively controlled gray mold disease of cucumber

**Figure 6:**
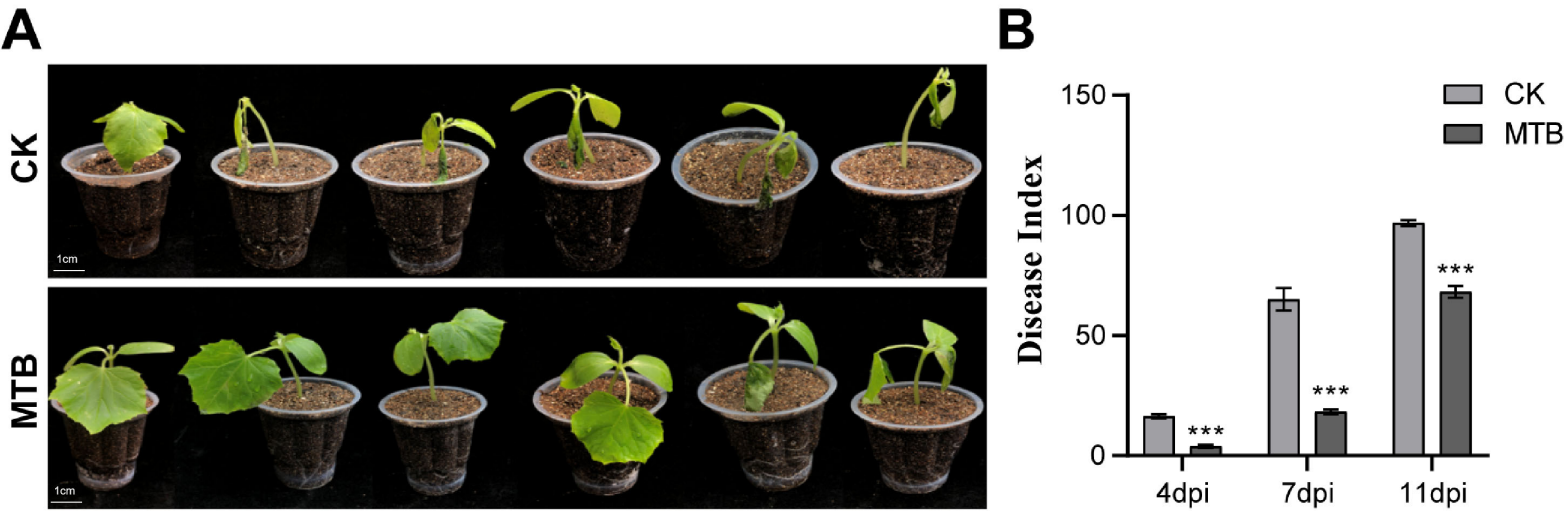
The pot control effect of MTB on cucumber gray mold disease. **(A)** Growth situation of the MTB treatment group and the control treatment 7 days after inoculation with *B. cinerea*. **(B)** Statistical chart of disease indices for different treatment groups on the 4th, 7th, and 11th dpi. Values are the mean ± standard deviation of three biological replicates. Significant differences were analyzed using Student’s *t*-test (*** *p*< 0.001). dpi, day-post inoculation.

A pot experiment was conducted to further evaluate the efficacy of MTB in managing cucumber gray mold disease caused by *B. cinerea*. The progression of the disease and the effectiveness of MTB were monitored over a period of 11 days, with disease index recorded to quantify the pathogen’s impact on plant health. On day 4, both the control and MTB-treated groups showed increasing water-soaked lesions on cucumber leaves, which gradually turned light brown with distinct edges and a thin layer of *B. cinerea* on the lesions. The disease index in the control group was 16.4 ± 0.8, while the MTB-treated group exhibited a significantly lower disease index of 3.9 ± 0.6. By day 7, the stems began to show symptoms, with a grayish-white surface and a dense *B. cinerea* layer. However, the MTB-treated group displayed a lighter disease condition compared to the control group (**Figure 6A**). The disease index for the control group was 65.1 ± 4.6, while the MTB-treated group had a disease index of 18.3 ± 1.0. By day 11, the lesions had encircled the stem, causing it to break and leading to plant death. The disease index for the control group reached 96.8 ± 1.2, whereas the MTB-treated group showed a disease index of 68.2 ± 2.5. The disease index in the MTB-treated group was significantly lower than in the control group at all time points (**Figure 6B**). During the experiment, some cucumber leaves exhibited typical hypersensitive response (HR) symptoms after inoculation with *Botrytis cinerea*. These symptoms were characterized by rapid localized cell death, forming distinct necrotic spots that were usually confined to the infection site with clear boundaries and no obvious expansion into surrounding tissues. This localized cell death is a self-defense mechanism of plants to restrict the spread of invading pathogens. Collectively, these results provide compelling evidence that MTB effectively controls cucumber gray mold disease by significantly reducing the disease index in treated plants compared to the control group.

### 3.7 Transcriptome analysis of MTB-treated cucumber leaves reveals key immune-related gene activation

**Figure 7:**
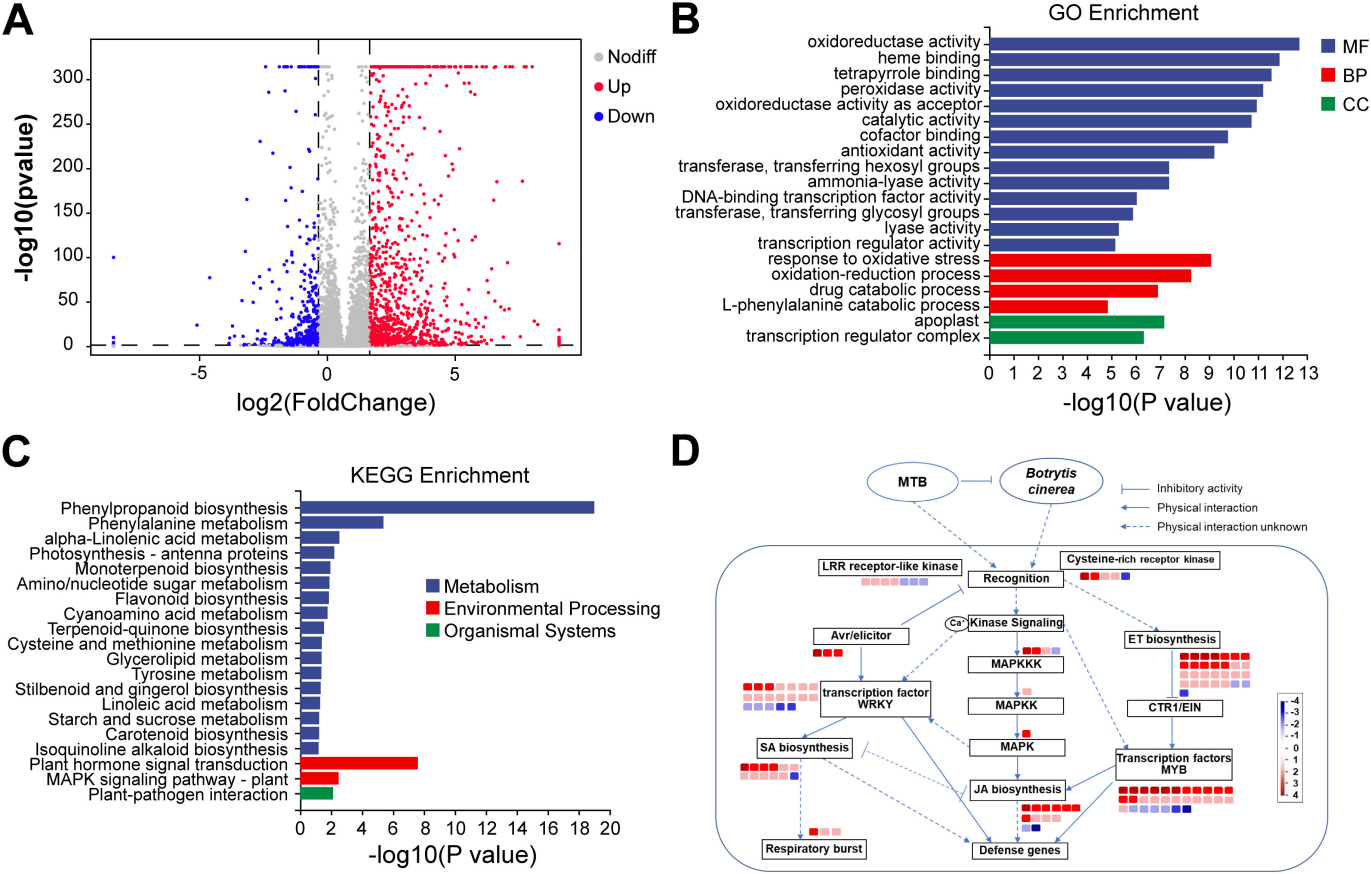
Comparative transcriptome analysis results. **(A)** Volcano plot of differentially expressed genes between MTB treatment and sterile water control groups. Each point in the plot represents a gene, with gray points indicating genes without significant differences, blue points indicating significantly downregulated genes, and red points indicating significantly upregulated genes. Points closer to the sides and the top of the plot show more significant differential expression. The horizontal axis represents the fold change in gene/transcript expression between the two samples, and the vertical axis represents the p-value, with |log2FoldChange| > 1 and a significance *P*-value< 0.05. **(B)** Top 20 metabolic pathways from the GO enrichment analysis of DEGs between MTB treatment and sterile water control groups. **(C)** Top 20 metabolic pathways from the KEGG enrichment analysis of DEGs between MTB treatment and sterile water control groups. **(D)** Immune regulation network under abiotic stress of DEGs in the MTB/CK comparison (MapMan).

To investigate the molecular mechanisms underlying MTB-induced resistance in cucumber against *B. cinerea*, we conducted a comparative transcriptome analysis of cucumber leaves treated with 0.02 mg/ml MTB and sterile water. A total of 1886 differentially expressed genes (DEGs) were identified, including 1337 upregulated genes and 549 downregulated genes (**Figure 7A**). Functional annotation of these DEGs revealed enrichment in categories related to molecular functions, such as “oxidoreductase activity,” and biological processes, including “oxidation-reduction processes” and “antioxidant response,” which are critical for plant defense against pathogens (**Figure 7B**).

KEGG pathway analysis highlighted the involvement of key pathways in MTB-induced resistance, including “Phenylpropanoid biosynthesis,” “Flavonoid biosynthesis,” and “Plant hormone signal transduction” (**Figure 7C**). Notably, the salicylic acid (SA), jasmonic acid (JA), and ethylene (ET) signaling pathways were significantly upregulated, with genes related to SA biosynthesis (e.g., PAL, SCMT, SOMT) and markers of SA signaling (e.g., PR-4, RGA2-like) being notably activated. Additionally, the JA signaling pathway, indicated by upregulation of LOX, AOS, and JMT genes, also played a critical role in MTB-induced defense responses. The ethylene pathway was similarly altered, with a marked increase in ethylene response factors (**Supplementary Table S1**).

Further analysis of DEGs involved in immune responses, including oxidoreductase, chitinase, pectinase genes as well as polygalacturonase-inhibiting genes (**Supplementary Table S2**). Moreover, some DEGs were involved in signaling pathways activation, for example, MAPK cascade kinases, receptor-like kinases (Cysteine-rich receptor-like kinase, LRR receptor-like kinase family protein, calcium-sensing receptor), avirulence factor rapid response proteins (Avr9/Cf-9 rapidly elicited protein, elicitor-responsive protein 1-like), and transcription factors (e.g., WRKY, NAC, MYB), thus achieving immune responses and forming hypersensitive necrosis reactions, a process closely related to the regulation of the SA signaling pathway (**Supplementary Table S3**). Additionally, MTB treatment can induce changes in the ET signaling pathway, with a large number of ethylene response factors being up regulated, which can further regulate the host’s immune response and the immune response mediated by the JA signaling pathway (**Figure 7D**). These findings indicate that MTB treatment activates multiple immune regulatory pathways, including SA, JA, and ET signaling, to enhance resistance against *B. cinerea*.

## 4 Discussion

*Botrytis cinerea* is a highly destructive pathogen that affects over 200 plant species worldwide, including numerous crops, fruits, and vegetables, leading to significant agricultural losses (Nambeesan et al., 2012; Yuan et al., 2024). Extensive research has focused on isolating, characterizing, and selecting effective antagonistic bacteria for the control of plant pathogens, including *B. cinerea* (Martínez-Absalón et al., 2014; Hunziker et al., 2015). *Bacillus amyloliquefaciens* strain GD4a, a beneficial bacterium isolated from switchgrass, produces a functional bacterial extracellular exudate (BEE) that significantly disrupts the pathogenicity of *B. cinerea* by inhibiting fungal conidium germination and hypha formation. This strain shows great potential in managing gray mold disease (GMD) in plants (Yang et al., 2024).For instance, a previous study identified two novel endophytic bacterial strains, *Pseudomonas stutzeri* and *Stenotrophomonas maltophilia*, which exhibited antagonistic activity against *B. cinerea* (Rojas-Solís et al., 2018). The antagonistic effects were attributed to volatile organic compounds (VOCs) produced by these bacteria, such as aldehydes, alcohols, esters, and sulfur compounds. Among the identified sulfur compounds, dimethyl disulfide (DMDS), known for its antimicrobial properties, and S-MTB (also known as MTB) were highlighted (Huang et al., 2012). However, the molecular mechanism of MTB against the plant pathogen *B. cinerea* is largely unknown. In this study, we focused on MTB’s potential for the prevention and treatment of cucumber gray mold disease caused by *B. cinerea*, and analyzing its function during plant-pathogen interaction. In addition, Bacillus proteolyticus OSUB18 significantly enhances Arabidopsis resistance to *Botrytis cinerea* by activating induced systemic resistance (ISR), which involves increasing callose deposition and ROS production, upregulating ISR-related genes, and promoting the generation of acetoin (Yang et al., 2023a).

Our results confirm that MTB exhibits potent inhibitory effects on *B. cinerea*, with a high concentration of MTB reducing pathogen colony growth by nearly 100%. This suggests that MTB could be a viable candidate for controlling cucumber gray mold disease, which is caused by *B. cinerea*. In our subsequent experiments, we explored the molecular mechanisms underlying MTB’s action by assessing its impact on plant immune responses. Gene expression analysis revealed significant upregulation of *PR1* and *PI1* in MTB-treated cucumber plants. These genes are markers of salicylic acid (SA) and jasmonic acid (JA) signaling pathways, respectively, indicating that MTB triggers both pathways. Transcriptome data further supported these findings, with key genes involved in SA synthesis, including *PAL* (phenylalanine ammonia-lyase) and *SCMT* (salicylate carboxyl methyltransferase), showing increased expression. PAL is the key enzyme that catalyzes the conversion of phenylalanine to trans-cinnamic acid, a precursor for SA biosynthesis (Chen et al., 2021). In a similar context, studies on strawberry plants treated with the endophytic yeast *Hanseniaspora uvarum* prior to *B. cinerea* inoculation demonstrated upregulation of several SA-related genes, including *PR1*, *PR5*, *PAL*, and *WRKY1*, within 12 to 24 hours post-treatment (Dou et al., 2019; Yang et al., 2023b). Furthermore, the expression of PR4, a marker gene for SA-induced defense responses, was significantly increased, indicating a robust SA-mediated defense response in MTB-treated plants. In Arabidopsis, the defense genes regulated by SA include *PR1*, *PR2* (β-1,3-glucanase), and *PR5* (thaumatin-like protein) (Truman et al., 2007). The defense genes induced by JA are mainly *PR3*, *PR4*, *PDF1.2*, and *LOX2* (Møller and Chua, 1999; Cheong et al., 2002).

In addition to SA signaling, our transcriptomic analysis revealed substantial upregulation of key genes in the JA biosynthesis pathway, including *LOX* (lipoxygenase), *AOS* (allene oxide synthase), *AOC* (allene oxide cyclase), *OPR3* (12-oxophytodienoate reductase), and *JMT* (jasmonic acid carboxyl methyltransferase). LOX catalyzes the first step in JA biosynthesis, confirming that MTB treatment stimulates JA production. The increased expression of OPR3 further supports the notion that MTB enhances JA signaling, which is crucial for plant defense, stress adaptation, and growth regulation (Schaller et al., 2000). The involvement of ethylene (ET) signaling in MTB-induced immunity was also evident, as many ERF (ethylene response factor) genes were significantly upregulated. ERF proteins, key transcription factors in ET signaling, play a critical role in plant immune responses (Fujimoto et al., 2011). Comparative transcriptome analysis revealed that a large number of ethylene response factor genes ERF were significantly up regulated, which is consistent with the experimental results. Clearly, in MTB treatment, SA, JA, and ET all participate in the immune regulation of plants.

Histopathological analysis of MTB-treated cucumber leaves revealed a marked reduction in necrotic lesions, with less cell damage compared to untreated controls. This protective effect was further corroborated by gene expression changes in enzymes involved in reactive oxygen species (ROS) detoxification, such as POD (peroxidase) and RBO (respiratory burst oxidase) (Hasanuzzaman et al., 2020). ROS accumulation is a hallmark of plant defense responses, and the upregulation of *POD* and *RBO* suggests that MTB treatment mitigates ROS-related damage, thereby reducing cell death and enhancing plant resistance. POD is particularly important in oxidative stress responses, as it helps detoxify ROS and contributes to cell wall fortification (Liu et al., 2024). Moreover, the significant upregulation of genes encoding chitinases, pectinases, and polygalacturonase inhibitors points to the activation of basal immune responses in MTB-treated plants, which are often regulated by JA signaling. These responses contribute to the reinforcement of plant cell walls and the inhibition of pathogen invasion (Morkunas and Gmerek, 2007). Our study also highlighted the role of MTB in modulating plant-pathogen interactions at the molecular level. In pathogen quantity assays, lower levels of *B. cinerea Actin* expression were detected in MTB-pretreated cucumber leaves, indicating reduced pathogen proliferation. This was confirmed in pot experiments, where MTB-treated plants exhibited lower disease index at both early and later stages of infection. These findings suggest that MTB acts as an elicitor that primes plant immunity, triggering both local and systemic defense responses.

The induction of a hypersensitive response (HR), characterized by a rapid cell death reaction at the site of pathogen infection, is a well-established feature of plant defense (Morel and Dangl, 1997) In this study, MTB’s recognition by plant receptors likely triggered HR through the activation of SA, JA, and ET signaling pathways. Notably, the activation of WRKY transcription factors, regulated by SA signaling, plays a crucial role in the transcriptional activation of defense genes. WRKY proteins are key regulators of plant immune responses and contribute to the fine-tuning of defense reactions (Kumar et al., 2020). Additionally, MTB’s interaction with pattern recognition receptors (PRRs), such as LRR and CRR kinases, may trigger the mitogen-activated protein kinase (MAPK) cascade, which amplifies stress signals and activates downstream defense responses.

In conclusion, our findings demonstrate that MTB effectively mitigates cucumber gray mold disease caused by *B. cinerea*. This is achieved through the activation of multiple immune signaling pathways, including SA, JA, and ET, which work synergistically to enhance plant defense. The modulation of key defense genes, the reduction in ROS accumulation, and the inhibition of pathogen growth collectively highlight MTB’s potential as a promising biocontrol agent for managing *B. cinerea* infections in cucumbers.

In conclusion, this study demonstrates that MTB significantly inhibits the growth and colonization of *B. cinerea* on cucumbers, both *in vitro* and *in vivo*. We investigate the anti-*B. cinerea* effects of MTB, providing novel insights into its potential as a biocontrol agent. *In vitro* assays showed a remarkable 98.6% inhibition of *B. cinerea* growth at a concentration of 2 mg/mL MTB, while *in vivo* experiments revealed a reduction in disease severity, suppression of infection, and alleviation of necrotic symptoms in cucumber leaves. Transcriptome analysis further elucidated the underlying mechanisms, highlighting MTB’s role in activating plant immune responses, particularly through the MAPK signaling pathway. MTB treatment resulted in upregulation of key defense genes, including *chitinase*, *pectinase*, and *lignin* biosynthesis genes, as well as modulating the SA, JA, and ET pathways. Notable increases in genes such as *POD*, *PAL*, *LOX*, and ethylene-responsive factors were observed, indicating MTB’s ability to strengthen plant immunity. These findings support the theory of induced resistance as an innovative strategy for controlling *B. cinerea*, offering promising directions for future research on enhancing plant disease resistance.

## Data Availability

The datasets presented in this study can be found in online repositories. The names of the repository/repositories and accession number(s) can be found in the article/**Supplementary Material**.

## Supplementary material

Supplementary Table 1 DEGs involved in hormone immune responses.

Supplementary Table 2 DEGs involved in resistance proteins.

Supplementary Table 3 DEGs involved in immune signaling pathways.

Supplementary Table 4 Primers used in this study.
