# Supplementary material for: Role of Methyl thiobutyrate to Botrytis cinerea on cucumber
Source: Front Plant Sci. 2025 Apr 8;16:1551274. doi: 10.3389/fpls.2025.1551274 (PMC12013339; doi:10.3389/fpls.2025.1551274)
Supplement: Supplementary Table 1 — DEGs involved in hormone immune responses. [file DataSheet1.zip › source data/RNA-seq raw data.docx]

The dataset supporting the conclusions of this article is available in the NCBI Sequence Read Archive repository, under accession number PRJNA1194377.

<https://www.ncbi.nlm.nih.gov/sra/?term=PRJNA1194377>
