# Supplementary material for: Role of Methyl thiobutyrate to Botrytis cinerea on cucumber
Source: Front Plant Sci. 2025 Apr 8;16:1551274. doi: 10.3389/fpls.2025.1551274 (PMC12013339; doi:10.3389/fpls.2025.1551274)
Supplement: Supplementary Table 1 — DEGs involved in hormone immune responses. [file DataSheet1.zip › source data/FIGURE S1.docx]

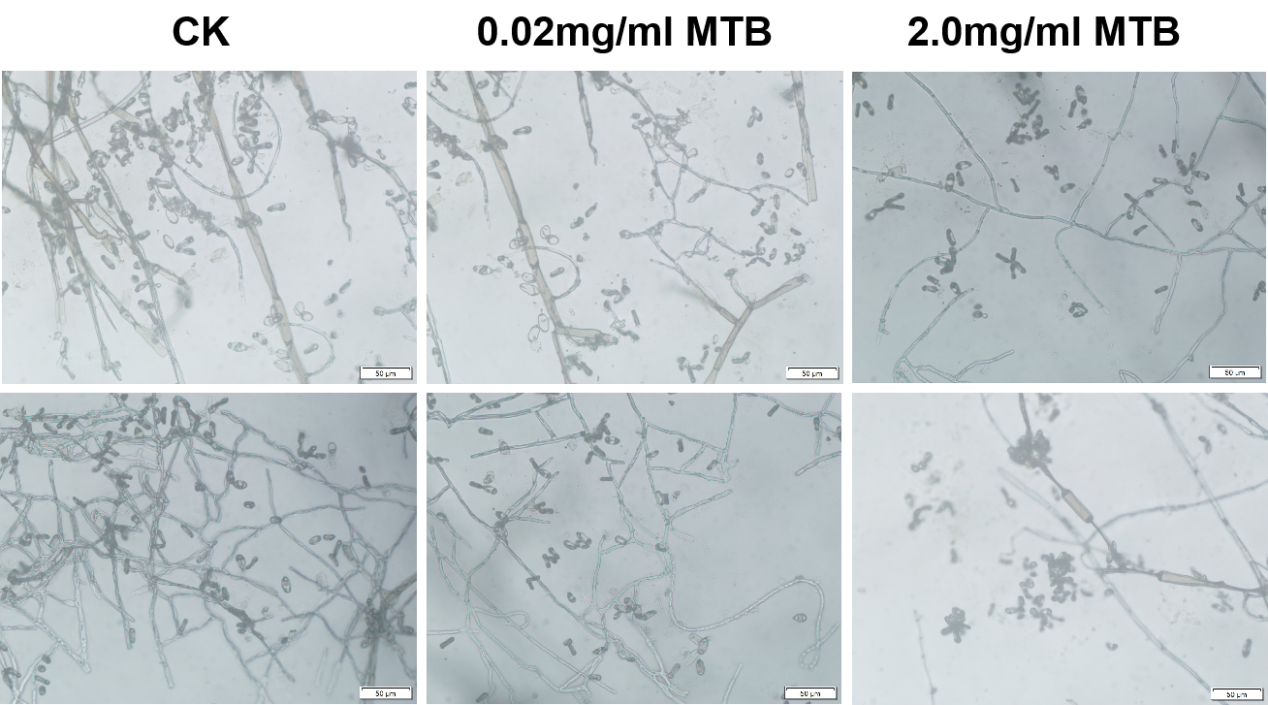


**FIGURE S1 ▏**Microscopic images of *B. cinerea* colonies on PDA plates treated with sterile water (as a control group), 0.02 mg/mL MTB, and 2 mg/mL MTB, respectively.
