# Supplementary figures and images for: Role of Methyl thiobutyrate to Botrytis cinerea on cucumber
Source: Front Plant Sci. 2025 Apr 8;16:1551274. doi: 10.3389/fpls.2025.1551274 (PMC12013339; doi:10.3389/fpls.2025.1551274)

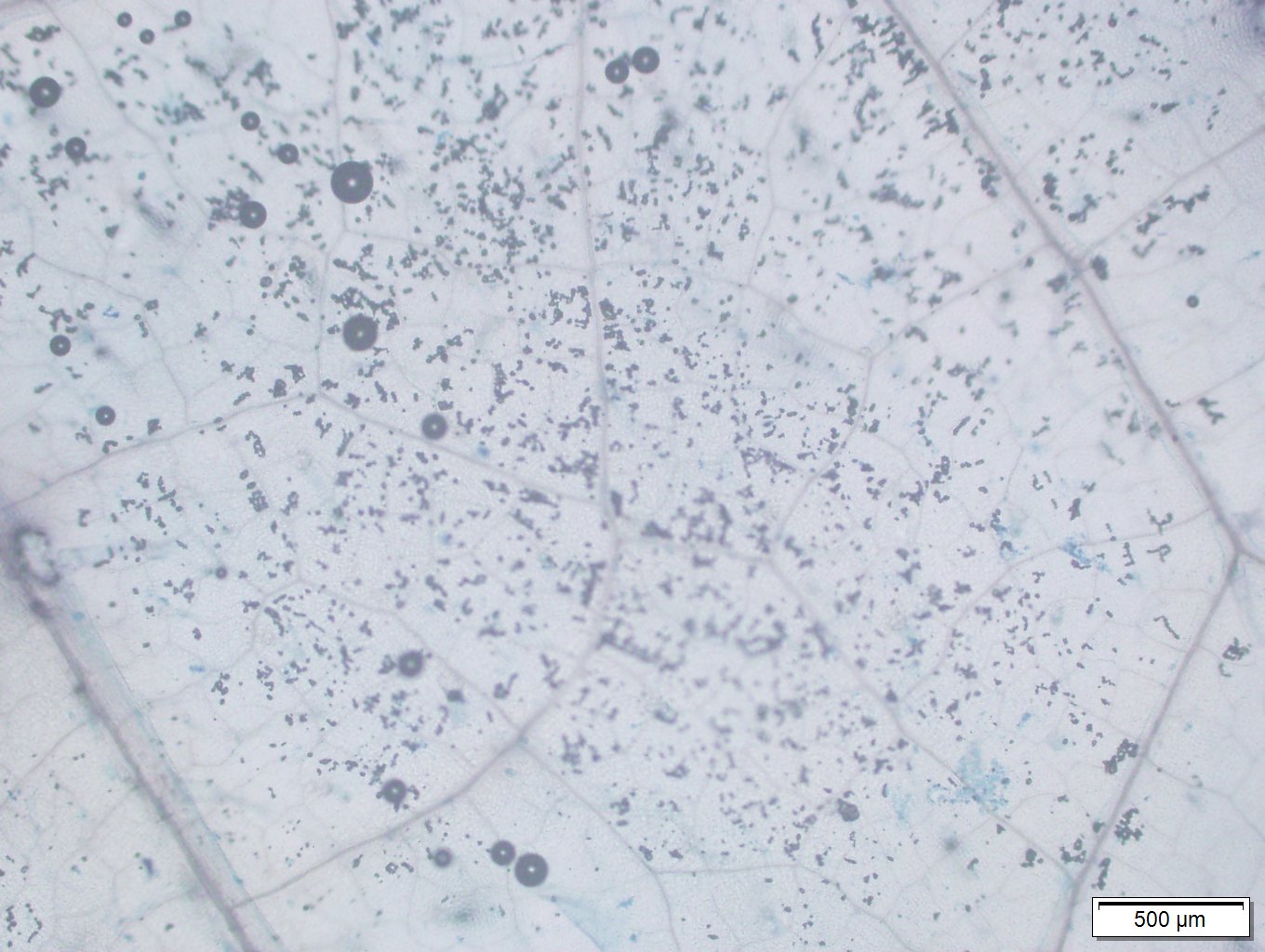

Supplement: Supplementary Table 1 — DEGs involved in hormone immune responses. [file DataSheet1.zip › source data/Microscope images/FIGURE 4/CK 1dpi/═╝╧±_7678.jpg]

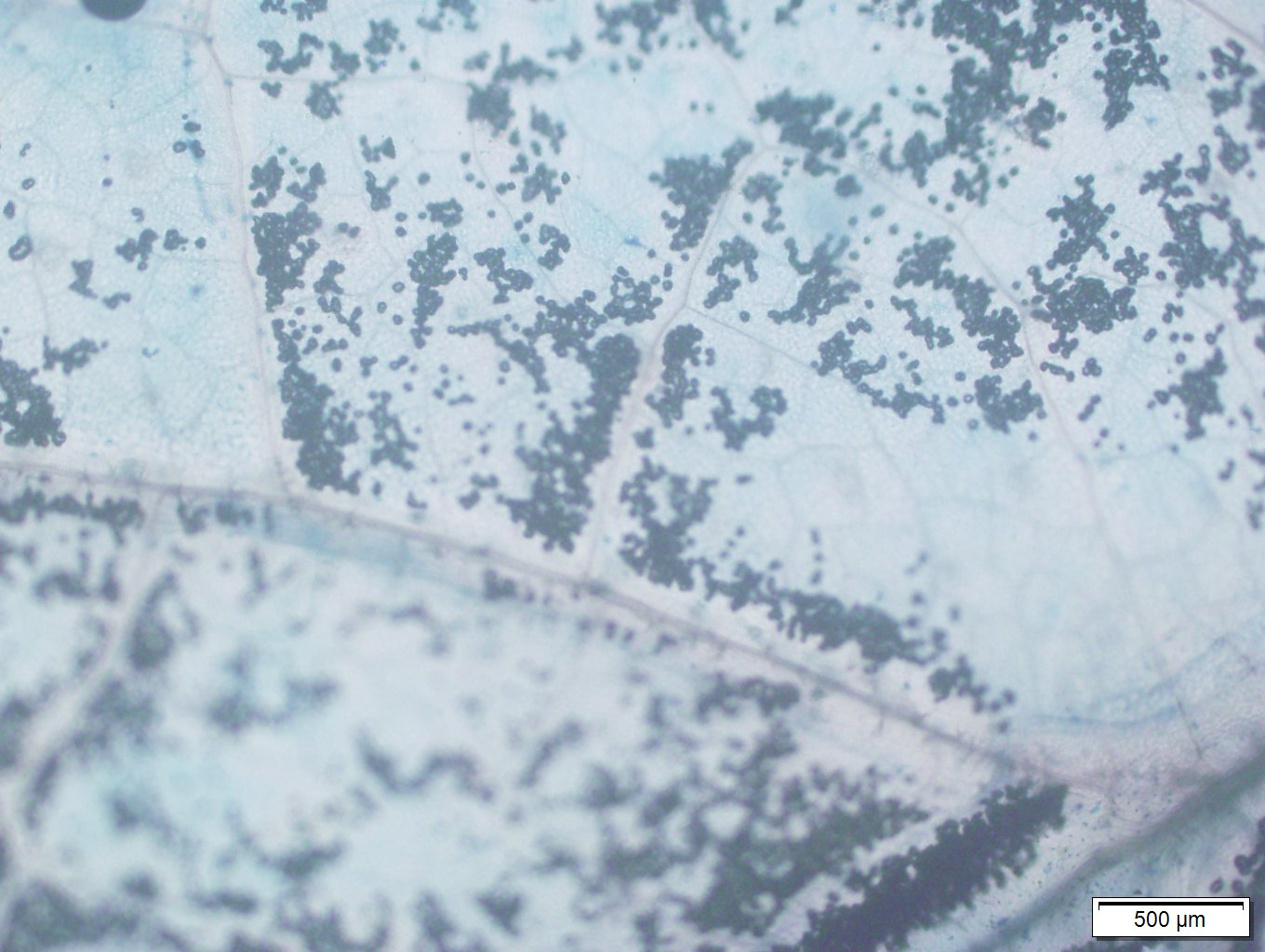

Supplement: Supplementary Table 1 — DEGs involved in hormone immune responses. [file DataSheet1.zip › source data/Microscope images/FIGURE 4/CK 3dpi/═╝╧±_7704.jpg]

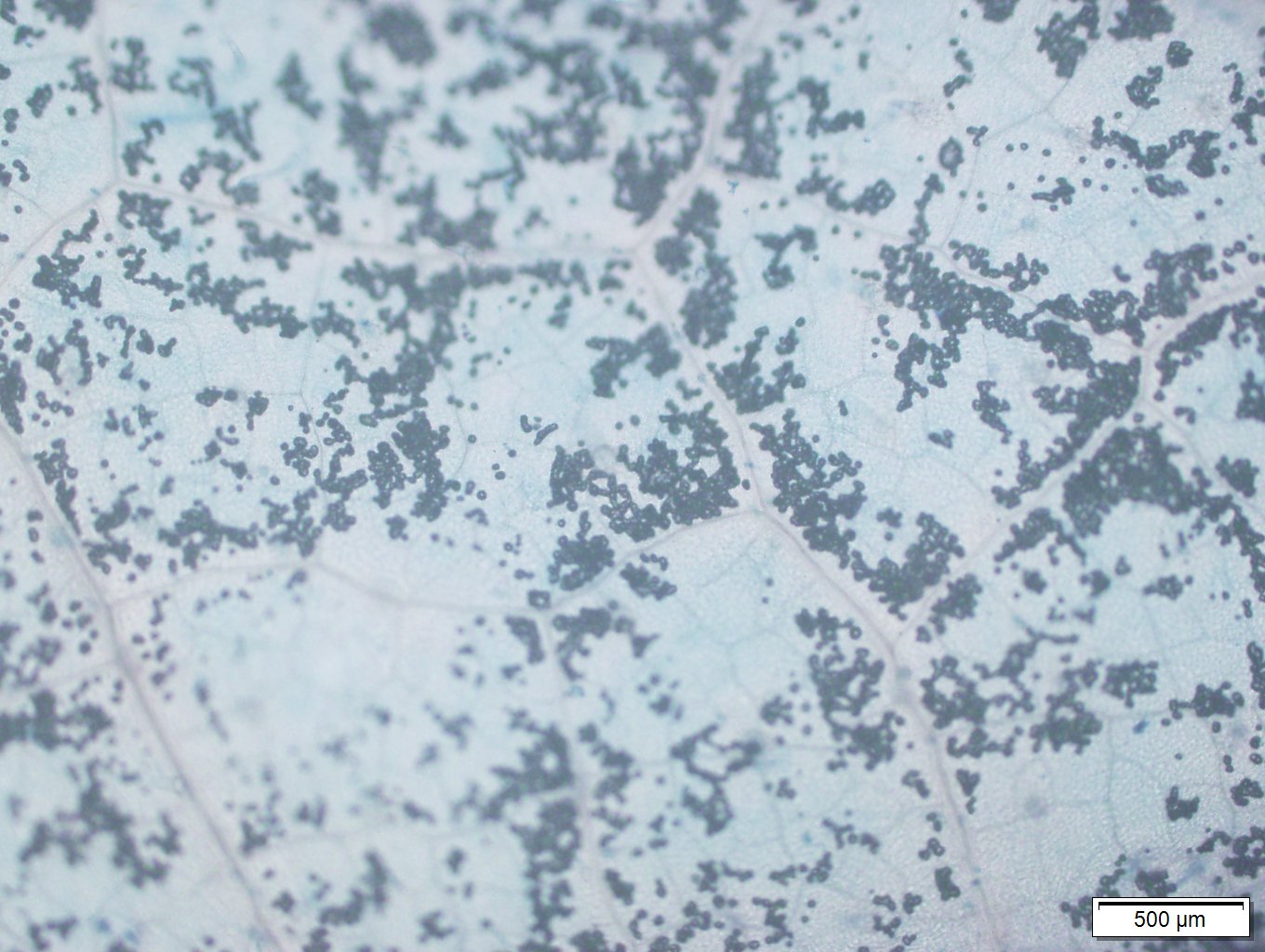

Supplement: Supplementary Table 1 — DEGs involved in hormone immune responses. [file DataSheet1.zip › source data/Microscope images/FIGURE 4/CK 7dpi/═╝╧±_7724.jpg]

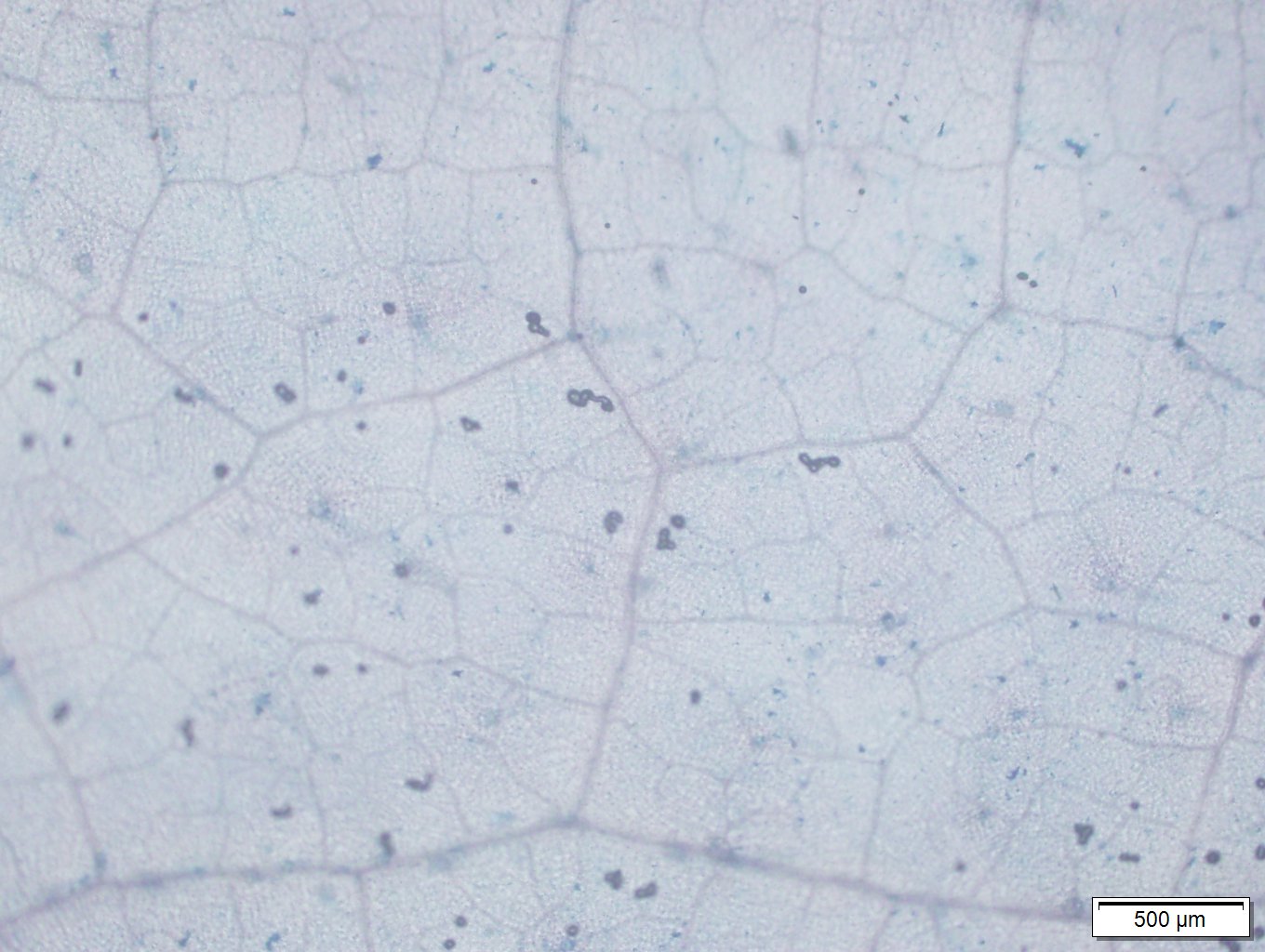

Supplement: Supplementary Table 1 — DEGs involved in hormone immune responses. [file DataSheet1.zip › source data/Microscope images/FIGURE 4/MTB 1dpi/═╝╧±_7687.jpg]

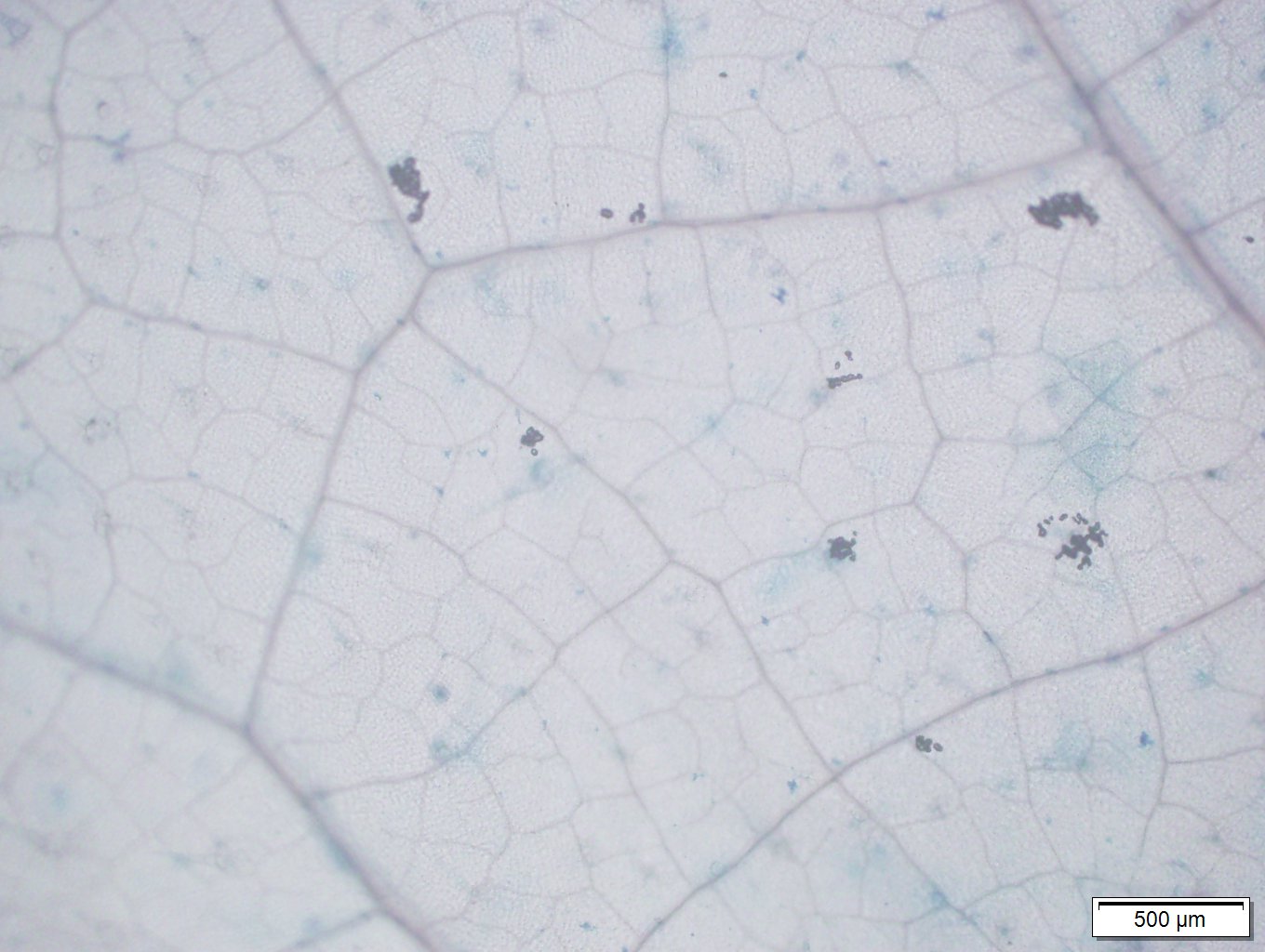

Supplement: Supplementary Table 1 — DEGs involved in hormone immune responses. [file DataSheet1.zip › source data/Microscope images/FIGURE 4/MTB 3dpi/═╝╧±_7716.jpg]

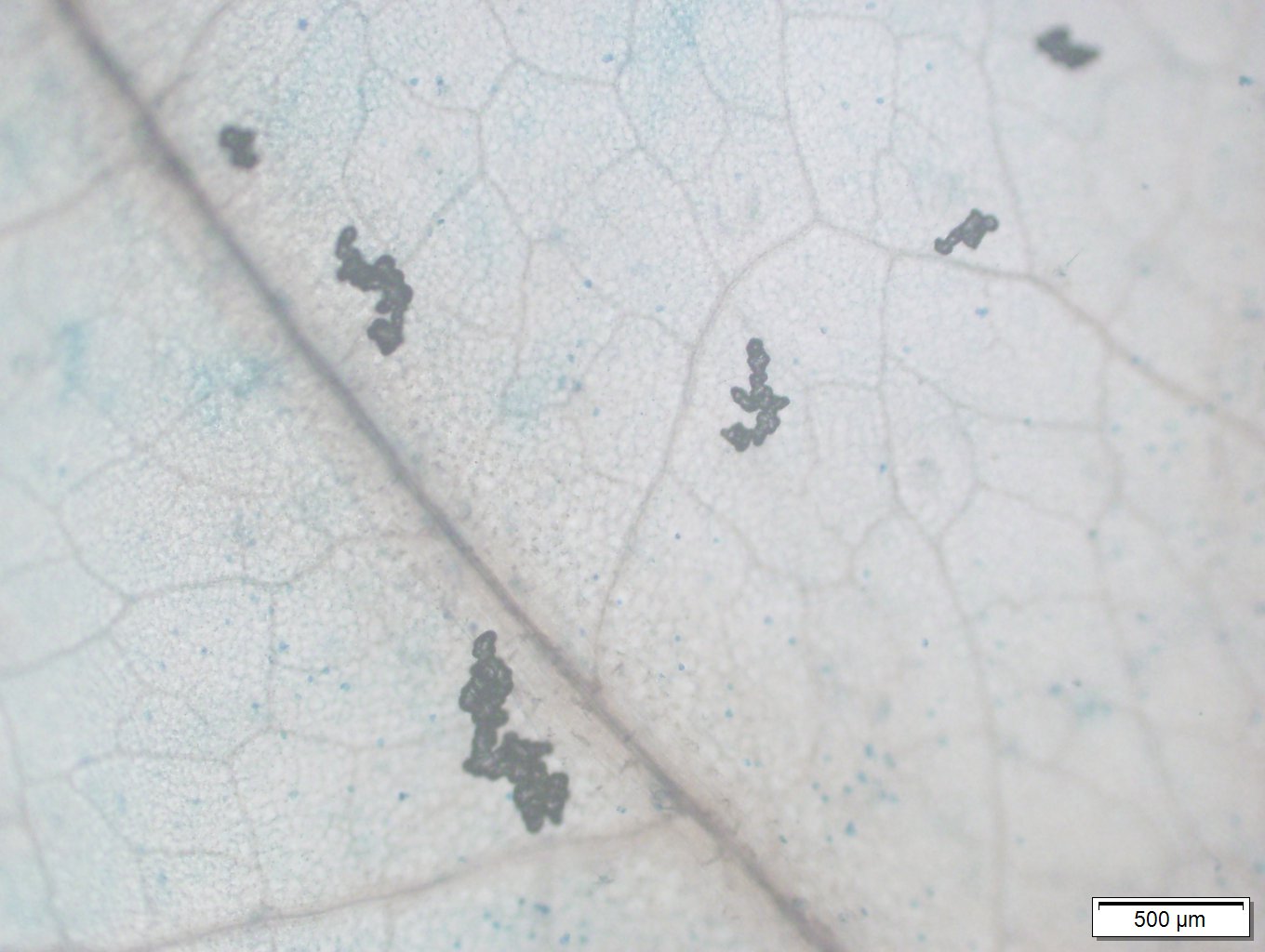

Supplement: Supplementary Table 1 — DEGs involved in hormone immune responses. [file DataSheet1.zip › source data/Microscope images/FIGURE 4/MTB 7dpi/═╝╧±_7737.jpg]
